# Analysis of associations between the *TLR3* SNPs rs3775291 and rs3775290 and COVID-19 in a cohort of professionals of Belém-PA, Brazil

**DOI:** 10.3389/fcimb.2023.1320701

**Published:** 2023-12-19

**Authors:** Marcos Jessé Abrahão Silva, Marcelo Cleyton da Silva Vieira, Alex Brito Souza, Everaldina Cordeiro dos Santos, Beatriz dos Reis Marcelino, Samir Mansour Moraes Casseb, Karla Valéria Batista Lima, Luana Nepomuceno Gondim Costa Lima

**Affiliations:** ^1^ Molecular Biology Laboratory, Bacteriology and Mycology Section (SABMI), Evandro Chagas Institute (IEC), Ananindeua, Brazil; ^2^ Molecular Biology Laboratory, Pathology Section (SEPAT), Evandro Chagas Institute (IEC), Ananindeua, Brazil

**Keywords:** *TLR3*, single nucleotide polymorphism, COVID-19, epidemiology, genetics

## Abstract

The objective of this article was to verify associations between the SNPs rs3775291 (Cytosine [C]>Thymine [T]) and rs3775290 (C>T) of *TLR3* in professionals from Health Institutions (HI) who worked during the first pandemic wave and COVID-19. A case-control study was carried out with workers from HI in Belém-PA, Brazil, divided into symptomatology groups (Asymptomatic-AS, *n*=91; and Symptomatic-SI, *n*=121), and severity groups, classified by Chest CT scan (symptomatic with lung involvement – SCP, *n*=34; symptomatic without lung involvement – SSP, *n*=8). Genotyping was performed by Sanger sequencing and statistical analysis was performed using the SPSS program. In the analysis of SNP rs3775291, the homozygous recessive genotype (T/T) was not found and the frequency of the mutant allele (T) was less than 2% in the cohort. For the rs3775290 SNP, the frequency of the mutant allele (T) was greater than 42% in the cohort. No significant associations were found for these SNPs in this cohort (N= 212 individuals). The scientific community and physicians can use these facts to find new methods of managing COVID-19.

## 1 Introduction

Coronavirus disease 2019 (COVID-19) is an infectious disease caused by severe acute respiratory syndrome coronavirus 2 (SARS-CoV-2), a Beta-coronavirus, member of the *Coronaviridae* family. This viral agent is capable of generating asymptomatic clinical conditions, symptomatic pulmonary and extrapulmonary (multisystem) manifestations, and even death (Silva et al., 2023a). Since its initial discovery in December 2019 in Wuhan province, China, this virus has already been linked to millions of fatalities worldwide and irreversible socioeconomic harm to mankind (World Health Organization, 2023). Determining host-associated risk factors is a valuable approach to understanding the causes behind the varying clinical outcomes of COVID-19 in people from various global populations (Abdelzaher et al., 2020; Russell et al., 2023).

The most common kind of genetic variation in the human genome is single nucleotide polymorphisms, or SNPs, among the genetic factors that have been investigated for correlation with COVID-19 (Liu et al., 2019). A SNP can impact every step of the synthesis and expression of a gene that produces a protein in the human body, potentially leading to structural, conformational, and functional alterations (Fadason et al., 2021). Given that the immune response that is triggered can alter and change the clinical course of an illness, single nucleotide polymorphisms (SNPs) in candidate genes, particularly those that encode proteins involved in the immune response, are very valuable in the identification of predictive biomarkers of prognosis and susceptibility to infectious diseases. sickness comparable to COVID-19 (Gagliano et al., 2019).

In this context, *Toll-like Receptors* (*TLRs*) are genes that encode transmembrane receptors of the same name (TLR1-10) present in mammals and are responsible for inducing the innate immune response by recognizing pathogen-associated molecular patterns (PAMPs) and molecular damage associated pathogens (DAMPs) through antigen presenting cells (APCs) (Aguirre-García et al., 2019). As pattern recognition receptors (PRRs), TLRs regulate the generation of cytokines and indirectly activate the adaptive immune system in addition to inducing the innate immune system. However, TLRs can potentially aggravate the inflammatory response (Kawai and Akira, 2010; Wang et al., 2015).

A growing body of evidence points to the significance of dysregulated TLR signaling in the genesis of infectious illnesses. Expression of TLR7 and TLR3 was observed in conjunction with lung inflammation caused by the respiratory syncytial virus (Huang et al., 2009). Influenza A virus (Guillot et al., 2005) and rhinovirus infection (Hewson et al., 2005) upregulated the expression of *TLR3*. PAMPs and DAMPs from viral etiological agents are the main molecules that TLR3, a transmembrane receptor, can identify (Alexopoulou et al., 2001). TLR3 recognizes dsRNA, a virus-specific molecular fingerprint. Although it can also encourage an excessively active and imbalanced immune response to infection, which is detrimental to the host and exacerbates the severe form of the disease, it is essential for triggering the antiviral state and stopping viral replication (Lester and Li, 2014).

*TLR3*, a gene with five exons (coding regions), is found on human chromosome 4q35.1. The 904 amino acid protein produced by this gene gets its name after it and is responsible for identifying the double-stranded (ds) RNA of infectious agents, which is a step in viral replication, in cellular endosomes (Ye et al., 2020). When dsRNA associated with viral infection is detected, it triggers the activation of the nuclear factor kappa beta (NF-kB) and the production of type I interferons (IFN-Is). Consequently, it strengthens the host’s defenses against several illnesses (CHEN et al., 2021). Numerous epithelial cells, such as fibroblasts, neurons, and immune system cells, have TLR3, which is expressed primarily in the placenta and pancreas (Lester and Li, 2014).

In this sense, the objective of this article was to identify possible associations between the SNPs rs3775291 and rs3775290 of *TLR3* and symptoms and severity for COVID-19 in a cohort of professionals who worked in the first pandemic wave in Belém-PA, Brazilian Amazon Region.

## 2 Material and methods

### 2.1 Study design and ethical considerations

The present research is categorized as observational with a case-control design and a quantitative analytical approach. It was done in agreement with the Strengthening the Reporting of Observational Studies in Epidemiology (STROBE) guidelines (Von Elm et al., 2014). All participants gave their informed permission (Free and Informed permission Form - TCLE) after the regional ethics committees’ approval of the study methodology. The University of Pará State’s Research Ethics Committee (CAAE: 38113620.1.0000.5174) accepted this work, which is associated with the study project: “Análise da resposta ao SARS-CoV-2 em relação aos achados radiológicos e/ou à susceptibilidade genética individual”, with opinion number: 6,124,862. This research was carried out according to the Declaration of Helsinki (PP, 1964) and Resolution No. 466/2012 of the Brazilian National Health Council (Brasil, 2012).

### 2.2 Sampling process and variables

In 10 healthcare centers in Belém-PA, Brazil (the Brazilian Amazon geographical region), the research was conducted. A total of 214 professionals who actively worked in medical centers that served patients with COVID-19 between April 1, 2021, and June 30, 2020—across the administrative, medical, and general services domains—were selected for inclusion in the research using convenience sampling. Due to a lack of established safety protocols, overcrowding in healthcare facilities, lack of masks, and failure to use the N95 mask, all employees of Brazilian healthcare facilities during this time were exposed to SARS-CoV-2 (according to the health vulnerability situation reported in some countries during the first pandemic wave, such as Brazil) (Martin-Delgado et al., 2020; Quigley et al., 2021; World Health Organization, 2021).

First, these professionals were divided into two groups (asymptomatic – AS and symptomatic – SI). Those in group 2 who performed chest computed tomography (CCT) were classified as symptomatic with pulmonary involvement (SCP, ≥ 10% in CCT data) or symptomatic without lung involvement (SSP, less than 10% lung involvement). The use of COVID-19 CCT to assess severity is recommended by the WHO and the Brazilian Ministry of Health (Choi et al., 2020; UNA-SUS, 2020). This methodology to divide the subjects of the cohort into groups according to COVID-19 parameters analyzed and present the variables used in the study has also been previously described in our other previously published study with another SNP analysis in the same cohort (Silva et al., 2023c).

Since COVID-19 was declared a pandemic, it was established that there was a disease criterion based on symptomatology related to two of the main suggestive symptoms of the infection were present and these symptoms were connected to the clinical features typical of the COVID-19 first wave of pandemics (dry cough, fever, or dyspnea) (López-Juárez et al., 2021; Carvajal et al., 2022).

### 2.3 Laboratory procedures

The period of sample collection was January 6, 2021, to March 30, 2022. Venipuncture-taken blood samples were stored at -20°C for use in subsequent laboratory operations at the Evandro Chagas Institute (IEC)’s Molecular Biology Laboratory – LABIMOL, Bacteriology and Mycology Section (SABMI). The Dneasy Blood & Tissue kit (QIAGEN^®^, Venlo, Netherlands) was used for DNA extractions and the manufacturer’s guidelines were followed. The potential correlation between the *TLR3* SNPs rs3775291 and rs3775290 and the severity or susceptibility to COVID-19 was assessed for each of these professions.

Information on *TLR3* SNPs (SNP identification - ID) was obtained from the National Center for Biotechnology Information’s (NCBI), dbSNP website (http://www.ncbi.nlm.nih.gov/snp/; accessed on October 10, 2020) (Sayers et al., 2023). Therefore, only one primer was designed for the use and amplification of these two SNPs, due to their proximity to the chromosome 4 locus, where the SNP rs3775291 is located on chr4:186082920 and the SNP rs3775290 is situated on chr4:186083063 (data of primer used in this study described in **Table 1**) (Sayers et al., 2023).

**Table 1:** Primer used for amplification of *TLR3* SNPs rs3775291 and rs3775290.

Gene	Strand type	Sequence (5’-3’)	Guidance	Annealing temperature	Fragment length	Binding site (5’)
*TLR3*	Forward (F)	CGGGCTTTTCAATGTGAGG	Sense	60°C	678 base pairs (bp).	186083063
	Reverse (R)	GCATAACAACTTAGCACGGCT	Antisense			

5’ binding sites of the TLR3 gene according to the reference sequence NC_000004.12 (NCBI).

The typing of these SNPs was carried out by sequencing through DNA amplification using primers for the Polymerase Chain Reaction - PCR, which were designed by the Primer3Plus v2.0 program (http://www.bioinformatics.nl/primer3plus/; accessed on April 20, 2022) (Untergasser et al., 2007) of the respective genomic regions deposited in GenBank (Benson et al., 2000).

PCR was performed with Platinum Taq DNA Polymerase, DNA-free (Invitrogen^®^, Thermo Fisher Scientific Corporation, Waltham, Massachusetts, USA) using the thermocycler: initial denaturation at 95°C for 1 minute, followed by 35 denaturation cycles of 95°C for 30 seconds, annealing at 60°C for 30 seconds and extension at 72°C for 1 minute, after that, final extension at 72°C for 10 minutes (Lisby, 1999). To see the amplified DNA fragments in a photodocumentation device, the amplified products were electrophoresed in a 2% agarose gel with 3.0 µL using Sybr Safe (Invitrogen^®^, Thermo Fisher Scientific Corporation, Waltham, Massachusetts, USA).

The EasyPure PCR Purification Kit (TransGen Biotech Co.^®^, Beijing, Beijing, China) was used to purify the PCR products according to the manufacturer’s instructions. The BigDye X-Terminator kit was used to sequence the DNA fragment on an ABI 3130 Genetic Analyzer sequencer (Applied Biosystems^®^, Life Technologies, Thermo Fisher Scientific Corporation, Waltham, MA, USA) and allowed SNP areas of interest to be visualized and analyzed using the Bioedit v7.2.5 program (Hall, 1999), with subsequent BLAST on the NCBI website (https://blast.ncbi.nlm.nih.gov/Blast.cgi/; accessed on October 01, 2023).

### 2.4 Statistical analysis and presentation of data

Laboratory data were organized in a database using Microsoft Office Access (Microsoft Corp.^®^, Redmond, Washington, USA), and information was visually represented with tables or graphs made with Microsoft Office Excel (Microsoft Corp.^®^, Redmond, Washington, USA). To confirm the relationships between variables structured in 2x2 tables, the observed proportions of the presence of the SNP within each research group were analyzed using G, two-tailed chi-square (χ 2), and Fisher’s exact test with the aid of the IBM SPSS Statistics v26.0 software (IBM Corp.^®^, Armonk, NY, USA). Using the odds ratio (OR) test with a 95% confidence interval (CI), the association between exposure and desired outcomes was determined. For statistical significance, probability (p) < was taken into consideration.

The chi-square test (χ^2^) with *p* < 0.001 as a cutoff point for significance level was performed to measure the agreement of each SNP with the Hardy-Weinberg Equilibrium (HWE) using the Arlequin v3.5.1.2 software (Schneider et al., 2000; Chen et al., 2017a). G*Power v3.1.9.7 software was used to measure sample size power using a Chi-square goodness-of-fit test (Kang, 2021).

## 3 Results

### 3.1 Sample size power, normality of variables, Hardy-Weinberg equilibrium and epidemiological characteristics from individuals of this Belém professional cohort

For the cohort population of symptomatic (N = 121) and asymptomatic (N = 91) people with COVID-19, with error probability α of 0.05 and effect size of 0.3, sample size power was determined using the chi-square goodness-of-fit. It is statistically acceptable that the actual real power (1-β error probability) is 0.94, which is greater than 0.80 (Kang, 2021). The study’s variables were categorized and non-parametrically ordered, with counts expressed as percentages and absolute values. There was agreement between the SNPs rs3775291 and rs3775290 and HWE (*p* = 0.91 and *p* = 0.41, respectively). The epidemiological characteristics of this cohort of professionals from Belém (N = 214) have already been analyzed in association with the symptoms and severity for COVID-19 in our previous study (Silva et al., 2023c).

### 3.2 Visual characterization of structural modifications in TLR3 protein caused by these SNPs investigated in this present case-control study

**Figure 1:**
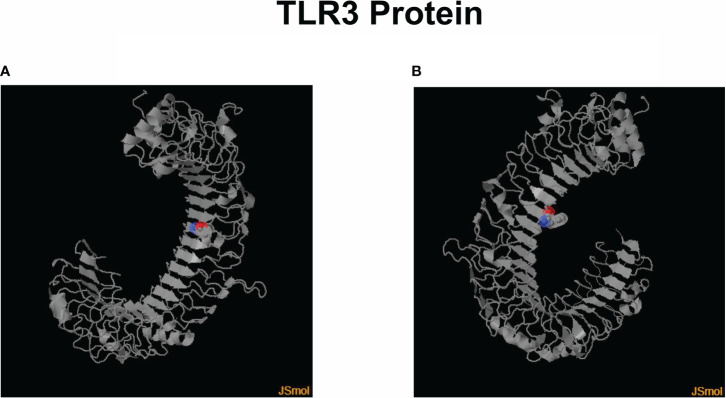
3D models of the human TLR3 protein with colored marking in the region of amino acid change through each SNP presence. **(A)** Leu412Phe change in its structure due to the presence of the SNP rs3775291. **(B)** Modification of Phe459Leu in its structure due to the presence of the SNP rs3775290. Created using Jmol v16.1.21 software (https://sourceforge.net/projects/jmol/; accessed on May 03, 2023).

Throughout the use of public data from the Protein Data Bank (PDB)/DSSP databases with entry UniProt O15455 (Human TLR3) and ID 3CIG, a visual representation of the target sites at which the SNPs rs3775291 and rs3775290, respectively, cause an exchange of codons in the amino acid triplet and therefore result in structural and functional modifications of the human TLR3 protein (**Figure 1**).

### 3.3 Genotyping data for *TLR3 SNPs* rs3775291 and rs3775290 in this Belém professional cohort (N = 212)

**Figure 2:**
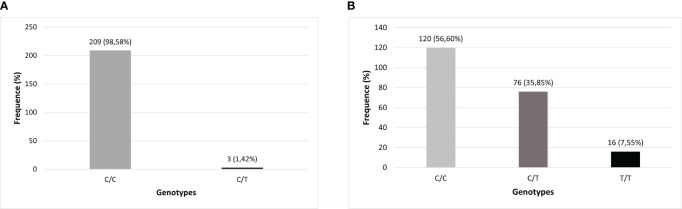
Genotypic distribution of *TLR3* polymorphisms in the studied population (N=212). **(A)** *TLR3* rs3775291 (C>T) genotypes; **(B)** *TLR3* rs3775290 (C>T) genotypes.

**Figure 3:**
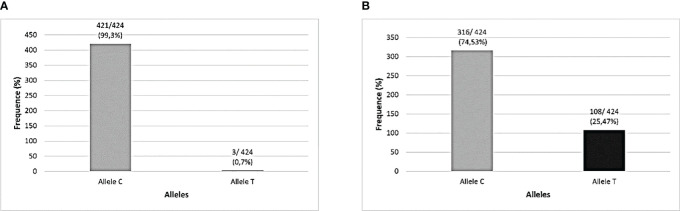
Allelic distribution of *TLR3* polymorphisms in the studied population (N=212). **(A)** *TLR3* rs3775291 (C>T) alleles; **(B)** *TLR3* rs3775290 (C>T) alleles.

There were technical difficulties in genotyping 2 samples from the cohort for the two *TLR3* SNPs investigated in the total number of samples, which is why they were removed from the analysis, both from subjects in the symptomatic group (one of them did not undergo a CCT test and the other belonged to the SCP group), resulting in a sample of 212 individuals for these analyzes. The distributions of genotypes and alleles found in this study cohort were described, respectively, in **Figures 2**, **3**.

### 3.4 Analysis of associations between *TLR3* SNPs (rs3775291 and rs3775290) and symptomatology and/or severity of COVID-19

**Table 2** characterizes the absolute and relative frequency of genotypes found for the *TLR3* SNPs evaluated in this cohort and the investigation of associations was carried out with respect to the AS and SI groups was carried out in relation to the wild-type allele of each variant. No significant associations were found for COVID-19 symptomatology and these *TLR3* SNPs in this cohort.

**Table 2:** Genotyping of study individuals for *TLR3* SNPs related to the symptomatology parameter.

Genotyping *n* (%)	AS (*n*=91) *n* (%)	SI (*n*= 121) *n* (%)	*p-*Value	
*TLR3* (rs3775291)				
C/C	90 (98.9%)	119 (98.3%)	*p* > 0.05	
C/T	1 (1.1%)	2 (1.7%)		
C **(wild allele)**	181 (99.5%)	240 (99.2%)	*p* > 0.05	
T	1 (0.5%)	2 (0.8%)		
*TLR3* (rs3775290)				
C/C	53 (58.2%)	67 (55.4%)	*p* > 0.05	
C/T	32 (35.2%)	44 (36.4%)		
T/T	6 (6.6%)	10 (8.3%)		
C/T +TT	38 (41.8%)	54 (44.6%)	*p* > 0.05	
C **(wild allele)**	138 (75.8%)	178 (73.6%)	*p* > 0.05	
T	44 (24.2%)	64 (26.4%)		

**Table 3** presents the absolute and relative frequency of genotypes and alleles found for each *TLR3* SNP and statistical analyzes of possible associations in relation to COVID-19 severity groups, based on the wild allele of these SNPs. No significant associations were found for COVID-19 severity and these *TLR3* SNPs in this cohort.

**Table 3:** Comparative genotyping analysis of this cohort for the *TLR3* SNP rs3775291 and rs3775290 related to COVID-19 severity.

Genotyping *n* (%)	AS (*n*=91) *n* (%)	SCP (*n*= 34) *n* (%)	SSP (*n*= 08)*n* (%)	*p-*Value (AS-SCP)	*p-*Value (AS-SSP)		*p-*Value (SCP-SSP)
*TLR3* (rs3775291)							
C/C	90 (98.9%)	34 (100%)	8 (100%)	*p* > 0.05	*p* > 0.05		–
C/T	1 (1.1%)	0	0				
C **(wild allele)**	181 (99.5%)	68 (100%)	16 (100%)	*p* > 0.05	*p* > 0.05		*p* > 0.05
T	1 (0.5%)	0	0				
*TLR3* (rs3775290)							
C/C	53 (58.2%)	18 (52.9%)	3 (37.5%)	*p* > 0.05	*p* > 0.05		*p* > 0.05
C/T	32 (35.2%)	14 (41.2%)	4 (50%)				
T/T	6 (6.6%)	2 (6.6%)	1 (12.5%)				
C/T +TT	38 (41.8%)	16 (47.1%)	5 (62.5%)	*p* > 0.05	*p* > 0.05		*p* > 0.05
C **(wild allele)**	138 (75.8%)	50 (73.5%)	10 (62.5%)	*p* > 0.05	*p* > 0.05		*p* > 0.05
T	44 (24.2%)	18 (26.5%)	6 (37.5%)				

## 4 Discussion

Covid-19 is considered a multisystemic and multifactorial disease (Abdelzaher et al., 2020). In this sense, several studies have already detected several host risk factors for COVID-19 in adults from different populations around the world, that is, particular conditions that increase the probability of becoming ill and, consequently, the appearance of symptoms, susceptibility and progression of severity. clinical picture of the disease in a systematically (Zhou et al., 2020). These factors can be exemplified as systemic arterial hypertension (SAH), cardiovascular diseases, obesity and demographic aspects, such as age and sex, in addition to the host’s own genetics (Rashedi et al., 2020).

Specific treatment for COVID-19 at a collective level is still a challenge for both the scientific community and health professionals, who must act in a joint and coordinated manner to articulate therapeutic strategies, which can range from types of options that attack the virus or those that target the host as a means of recovery and clinical restoration (Li et al., 2021; Yin et al., 2021).

Among the types of treatment for viral clearance, there are: antiviral agents, polymerase inhibitor medications, inhibitors of nucleoside and nucleotide reverse transcriptase, entry and uncoating inhibitors and others. In relation to the host as the main target, the main techniques used are: neutralizing antibody therapy, convalescent plasma, neutralizing monoclonal and polyclonal antibody therapies, Janus-kinase inhibitors, steroids, glucocorticoids, and others (Yuan et al., 2023).

Some of these medications, especially those that are conditioned to benefit the individual’s inflammatory clinical condition, take into account the activation of several molecular pathways in the nucleus of cells, which enable viral recognition of the host in the cell affected by PRRs (such as TLR3) in APCs (such as macrophages), the secretion of inflammatory mediators (such as IL-6, TNF-alpha, IFN-I and IFN-III) to condition the patient’s condition. In this sense, focusing treatment on PRRs, such as TLR3, through anti-TLR therapies generates inhibition and reduction of the formation of inflammatory cascade pathways (such as Janus Kinases), making it impossible for the patient to form a hyperinflammatory state (Karki et al., 2021). Therefore, SNPs rs3775291 and rs3775290 of the *TLR3* gene were genotyped in this cohort and their frequencies compared between the study groups for symptomatology and severity of COVID-19.

Although RT-PCR is the gold standard test for diagnosing COVID-19, Chest Computed Tomography (CCT) can be used to assist in the assessment and monitoring of lung lesions in these patients (Ai et al., 2020; Choi et al., 2020; Simpson et al., 2020). Therefore, the chest tomographic results of the individuals in the cohort were used to compare the severity of the disease in relation to epidemiological data and for genetic analysis with each *TLR3* SNP.

TLR3 is capable of inducing an antiviral immune response to both RNA and DNA viruses (Vercammen et al., 2008). The TLR3 subtype is believed to be the most clinically significant TLR that has been shown to react to coronaviruses (Totura et al., 2015). Following type II pneumocyte endocytosis of SARS-CoV-2 in the lungs, viral nucleotides are delivered to endosomes, including TLR3 and TLR7 (Grassin-Delyle et al., 2020). TLR3 functions by stimulating IRF-3 to create the cytokines interleukin (IL)-1, IL-1β, IL-4, IL-6, IFN-α, and IFN-β within the first twenty-four hours following SARS-CoV-2 infection. Next, TLR3 initiates the transduction pathway of NF-kB, resulting in the discharge of inflammatory cytokines (Bortolotti et al., 2021). In order to prevent SARS-CoV-2 infection, multi-epitope peptide vaccines (B cell and T cell epitopes) have been created that can bind to human TLR3 efficiently and generate a sufficiently strong enough immune response (Kalita et al., 2020).

The structure and function of TLR3 have been found to change due to polymorphisms, such as the rs377591 and rs3775290 SNPs, which can alter how the immune system responds to viruses (Ishizaki et al., 2008). *TLR3* SNP rs3775291, also known as L412F, is a non-synonymous (Cytosine to Thymine, C > T) missense mutation in exon 4, which changes the amino acid codon from Leucine (Leu) to Phenylalanine (Phe) at residue 412 and causes this receptor to become less active in the human body (Habibabadi et al., 2020). This variant was first reported in scientific records in 2005 (Hoffjan et al., 2005). This SNP has no effect on the amount of *TLR3* transcript, but it has been shown to reduce the ability of TLR3 to bind dsRNA and cause lower signaling activity (Barkhash et al., 2013).

Nuclear factor-κappa Beta (NF-kB) activation by approximately 50%, compared to leucine-containing TLR3 (C allele). The presence of 412Phe destabilizes the solenoid protein structure, which impacts the glycosylation potential of the nearby Asn413 residue (which has been demonstrated to be attached to N-acetyl glucosamines). This region is crucial for the dimerization of the domain in the membrane and forms the TLR3 receptor’s ectodomain (Redondo et al., 2022b).

The SNP rs3775290, also called F459L, consists of a non-synonymous and missense mutation, also present in exon 4, consisting of the change from Cytosine to Thymine (C>T). This *TLR3* genetic variant had its first association analysis in 2009 (Hwang et al., 2009). The mutation causes an amino acid change from Phe to Leu at position 459 of the protein (Phe459Leu), altering the ectodomain of TLR3, which negatively impacts the interaction between ligand and receptor [76] on the efficacy of signal transduction in the TLR3 (Mandal et al., 2012) and, therefore, results in a shortened immune response (Fischer et al., 2018).

The frequency of the mutant allele (T) of SNP rs3775291 previously reported by several studies registered at the National Center for Biotechnology Information (NCBI) in Latin American populations was very different from that found in the present research in a cohort of the Amazonian population in Brazil. The Latin American frequency of the presence of this SNP mutant allele varies between 0 and 28%, while in the cohort of this study, only approximately 2% of the (T) allele was found (National Center of Biotechnology Information, 2023b; Silva et al., 2023b). This distribution of alleles related to the genotyping of this SNP in the Amazonian cohort was different even in relation to other research published in other Brazilian regions (Santos et al., 2019; Singh and Samani, 2022). This may indicate that this genetic region is well conserved in this population.

Regarding the frequency of the presence of the variant allele (T) of the SNP rs3775290, its occurrence registered in the NCBI for Latin Americans can vary from 0 to 13%, however, in the present study, around 44% of our cohort presented the mutant allele. This represented an atypical finding also for this *TLR3 SNP* in this Amazonian population, as its presence was much higher than expected (National Center of Biotechnology Information, 2023a). Furthermore, even among Brazilian regions, it is higher than that already identified in a population from the Brazilian Northeast Region, in the study by Santos et al. (2023) for this SNP (28% of individuals in the Recife cohort) (Santos et al., 2023).

These results suggest that the genetic, epigenetic, and environmental factors that influence the genotypic profile of the Amazonian population differ significantly from those that prevail in the rest of Brazil and Latin America. The impact of the genetic origin phenomena and the unique characteristics of these people’s lifestyles may help to explain this case (Souza et al., 2019). These factors may be related to several elements that surround the host, such as those related to nutrition, body physiology, ethnic miscegenation, and others (Garg et al., 2020; Xie and Zhu, 2020; van der Made et al., 2022). They may also be associated with a predisposition to comorbidities, as in the cases of SNPs in other genes with higher frequency in this population associated with a worse prognosis of chronic conditions (such as the SNP rs7903146 of the *TCF7L2* gene associated with a worse prognosis of type 2 Diabetes Mellitus [DM2] in population from Southern Brazilian Region), which may be more prevalent in this population and influence the worse clinical evolution of COVID-19 (Assmann et al., 2014; Bastos et al., 2021).

Regarding the literary scenario on associations between the investigated *TLR3* SNPs and infections, several studies have already linked the rs3775291 SNP with infectious diseases (Lee et al., 2013; Chen et al., 2017b; Santos et al., 2019; Posadas-Mondragón et al., 2020; Redondo et al., 2022a). A recent meta-analysis by (Silva et al., 2023a; Silva et al., 2023b) summarized data found by primary research in several countries involving this SNP and infectious diseases. As a result, the authors found that the mutant allele (T) was linked to a 0.16-fold increased risk of virus infection worldwide (Silva et al., 2023b). One of the infectious diseases most studied to look for associations with this SNP was hepatitis B, caused by HBV (a type of DNA chain virus) (Wu et al., 2007; Li and Zheng, 2013; Geng et al., 2016).

Based on free global genomic datasets from worldwide populations, Dhangadamajhi and Routet (2021) investigated the likely association between the *TLR3* SNP rs3775291 and COVID-19 and concluded that the SNP is linked to susceptibility to disease and death (Dhangadamajhi and Rout, 2021). Although errors were found in the study, Pati et al. (2021) published these details so that the scientific community can thoroughly evaluate the conclusions of the previous article (Pati et al., 2021). Another case study with a five-year-old Brazilian vulnerable to hepatitis C, looked for associations between this SNP and the worst prognosis of COVID-19, however, the mutant allele (T) was not found in this patient (Pessoa et al., 2021). Therefore, as in both studies reported above, in this present research, both genotypic and allelic investigation for the *TLR3* SNP rs3775291 did not demonstrate a significant association with symptoms and/or severity of the disease.

Regarding the SNP rs3775290, several studies have been carried out to identify its association with infectious diseases and, in this sense, there are several similar studies between Hepatitis B and C. HCV, unlike HBV, is a sense positive ssRNA virus (+), as well as SARS-CoV-2. Kupffer cells and liver stellate cells (HSCs) which are found in the liver, express TLRs (such as TLR3) and are crucial in the development of liver fibrosis and HCV infection (Zayed et al., 2017). When HCV dsRNA activates *TLR3*, IFN-β is produced, which can prevent replication of viral infection (Broering et al., 2008).

Regarding to COVID-19, to date, only one case-control epidemiological study has been carried out to assess the association between the disease and the *TLR3* rs3775290 SNP. Case-control study by Alseoudy et al. (2022) sought to perform a comparative analysis in an Egyptian population between this SNP and the prognosis and susceptibility to COVID-19 pneumonia that accompany SARS-CoV-2 infection. They showed that the presence of the mutant (T) allele of the rs3775290 SNP in this population was associated with an increased risk of COVID-19 pneumonia and increased mortality from this related pneumonia (Alseoudy et al., 2022). On the other hand, in the case of the present study, no significant associations were found for SNP rs3775290 and COVID-19 in professionals.

Since genetically based phenomena and environmental factors are unique to each different population group, which collectively cause immunogenetic heterogeneity, the results of SNP association can differ from one community to another (Doetschman, 2009; van der Made et al., 2022). To adequately visualize the epidemiological and genetic characteristics of COVID-19 and the effects they cause, more research is needed in this area of study. Furthermore, more studies are needed to complement the data already collected on *TLR3 SNPs* in Brazilian regions. More SNPs in the *TLR3* gene should be examined in relation to clinical symptoms and severity in this population, or the same SNPs in different cohorts and with larger sample sizes.

One of the limitations of the study was that for COVID-19 test, as well as Personal Protective Equipment or Collective Protective Equipment (CPEs/PPEs), was difficult to obtain and in small quantity during the initial wave in several countries, mainly in developing countries (such as Brazil), so many professionals did not take the test at that time (Cotrin et al., 2020; El-Sokkary et al., 2021). The dyspnea symptom was removed from a potential severity rating because it was difficult to categorize it as a symptom of COVID-19 or as a psychological element, as it was not possible to obtain CT data from the entire cohort. Instead, reports from questionnaire participants were only considered at the conclusion of the analysis. As the data we analyzed were based on a questionnaire, it also had memory limitations. Our investigation, however, is limited to the first wave and so we believe that since it was such a tragic and crucial period in recent history, individuals still remember what happened at that time (Almeida et al., 2020; Miguel‐Puga et al., 2021).

## 5 Conclusions

TLR3 provides a potent antiviral immune function in the human host, including against SARS-CoV-2 infection. Therefore, the comparative analysis of the presence of missense SNPs rs3775291 (C>T) and rs3775290 (C>T) of *TLR3* gene, with already proven roles in reducing the performance of this immune receptor, are of great immunogenetic interest for evaluating interventional health measures against COVID-19. In this present study cohort, for SNP rs3775291, the homozygous recessive genotype (T/T) was not found, and the frequency of the mutant allele (T) was less than 2% in the cohort. For the rs3775290 SNP, the frequency of the mutant allele (T) was greater than 42% in the cohort. In this cohort, no significant relationships were discovered between these SNPs and COVID-19.

To find new methods to contain and to combat the disease, scientists and medicine can benefit from these present observed data to try to investigate other host risk factors for the disease. This study is especially valuable from the perspective of those working in the clinical-hospital environment against COVID-19.

## Data availability statement

The datasets presented in this study can be found in online repositories. The names of the repository/repositories and accession number(s) can be found in the article/supplementary material.

## Ethics statement

Informed consent was obtained from all subjects involved in the study. Approved by the Research Ethics Committee - CEP of the University of Pará State (UEPA), Brazil under opinion number 6,124,862.

## Author contributions

MJAS: Conceptualization, Methodology, Data curation, Formal analysis, Investigation, Writing – original draft, and Visualization. MV: Data curation, Formal Analysis, Investigation, Validation, Writing – original draft. AS: Data curation, Formal Analysis, Investigation, Methodology, Writing – original draft. ED: Data curation, Formal Analysis, Investigation, Methodology, Writing – original draft. BM: Data curation, Resources, Software, Writing – original draft. SC: Supervision, Visualization, Writing – review & editing. KL: Supervision, Visualization, Writing – review & editing. LL: Conceptualization, Investigation, Project administration, Supervision, Visualization, Writing – review & editing.
